# Glycosides of Nadifloxacin—Synthesis and Antibacterial Activities against Methicillin-Resistant *Staphylococcus aureus*

**DOI:** 10.3390/molecules27051504

**Published:** 2022-02-23

**Authors:** Mark Hutchins, Richard A. Bovill, Peter J. Stephens, John A. Brazier, Helen M. I. Osborn

**Affiliations:** 1 ThermoFisher Scientific, Wade Road, Basingstoke RG24 8PW, Hampshire, UK; 2 Reading School of Pharmacy, University of Reading, Whiteknights, Reading RG6 6AD, Berkshire, UK

**Keywords:** *Staphylococcus aureus*, MRSA, nadifloxacin, glycoside

## Abstract

The increase in the number of bacteria that are resistant to multiple antibiotics poses a serious clinical problem that threatens the health of humans worldwide. Nadifloxacin (**1**) is a highly potent antibacterial agent with broad-spectrum activity. However, its poor aqueous solubility has limited its use to topical applications. To increase its solubility, it was glycosylated herein to form a range of *trans*-linked (**3a-e**) and *cis*-linked (**7a,b**) glycosides, each of which was prepared and purified to afford single anomers. The seven glycoside derivatives (**3a-e, 7a,b**) were examined for potency against eight strains of *S. aureus*, four of which were methicillin-resistant. Although less potent than free nadifloxacin (**1**), the α-L-arabinofuransoside (**3a**) was effective against all strains that were tested (minimum inhibitory concentrations of 1–8 μg/mL compared to 0.1–0.25 μg/mL for nadifloxacin), demonstrating the potential of this glycoside as an antibacterial agent. Estimation of Log P as well as observations made during preparation of these compounds reveal that the solubilities of the glycosides were greatly improved compared with nadifloxacin (**1**), raising the prospect of its use in oral applications.

## 1. Introduction

Staphylococci are commonly found in the environment and are major colonizers of human skin [1,2]. *Staphylococus aureus*, the major human pathogen of the genus, is mostly harmless, but in certain situations it can cause severe illness, such as endocarditis, pneumonia, sepsis and toxic shock syndrome [3,4]. Infections were initially treated with benzylpenicillin, but by the late 1950s, resistant strains of the bacteria that produced β-lactamase were increasingly being isolated. In 1959, the β-lactamase-resistant antibiotic methicillin was introduced, but since then there has been a steady increase in the prevalence of methicillin-resistant strains of bacteria, and last-resort drugs such as vancomycin are increasingly used to treat infections [5,6]. The isolation of strains with reduced susceptibility to this drug, termed vancomycin-intermediate-resistant *S. aureus* (VISA), has raised the prospect of there being no antibacterial therapy for such strains [7].

**Scheme 1:**
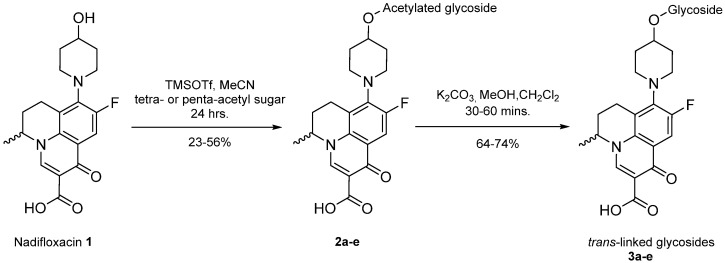
Synthesis of *trans*-linked nadifloxacin glycosides, specifically α-l-arabinofuranoside (**3a**), β-d-galactopyranoside (**3b**), β-d-glucopyranoside (**3c**), α-d-mannopyranoside (**3d**) and β-d-xylofuranoside (**3e**), from the respective protected intermediates (**2a-e**).

Nadifloxacin (**1**) (Scheme 1) is a broad-spectrum fluoroquinolone antibiotic that demonstrates high potency against aerobic Gram-positive and Gram-negative organisms and anaerobes. It has also been shown to be highly effective against methicillin-resistant *Staphylococcus aureus*, and low incidence of resistance is noted [8,9]. Unfortunately, nadifloxacin is poorly soluble in water and can only be used medicinally in topical ointments to treat acne and other skin infections [10,11]. Many attempts have been made to increase the aqueous solubility of nadifloxacin and thus enable it to be used in oral administration, for example by forming carboxylate salts, esters and peptides and by incorporating the drug in microemulsions and dendrimers [12,13,14,15,16,17]. However, the production of a suitable commercial derivative has so far been unsuccessful.

An alternative approach to improve the solubility of nadifloxacin is to glycosylate the free hydroxyl group on the piperidine ring (Scheme 1). Since this hydroxyl group is required for nadifloxacin to have antibacterial properties, masking of this position by formation of a glycoside would serve a dual purpose. Firstly, it would increase the aqueous solubility of nadifloxacin as required. Secondly, it would afford glycosides that would be less toxic than nadifloxacin itself. Specific bacteria that express the corresponding glycosidase enzyme would, however, be able to hydrolyse the glycosides to afford nadifloxacin, potentially affording targeted treatments for specific bacterial infections [18,19,20,21]. This approach was explored herein by synthesising and testing seven different nadifloxacin glycosides (**3a-e, 7a,b**) prepared from racemic nadifloxacin. The minimum inhibitory concentration (MIC) of each glycoside was then determined by means of a doubling dilution method.

## 2. Results and Discussion

To prepare the different anomers of the nadifloxacin glycosides, two different synthetic approaches were used. The *trans*-linked nadifloxacin glycosides, specifically α-l-arabinofuranoside (**3a**), β-d-galactopyranoside (**3b**), β-d-glucopyranoside (**3c**), α-d-mannopyranoside (**3d**) and β-d-xylofuranoside (**3e**), were synthesised by reacting nadifloxacin (**1**) with the *O*-acetylated glycosides in the presence of 10 molar equivalents of trimethylsilyl triflate (TMSOTf) in anhydrous acetonitrile (Scheme 1). The glycosides (**2a-2e**) were deprotected by treatment with potassium carbonate in methanol and then purified by adjusting the pH of the crude deprotection mixture to about 3.0 with formic acid and eluting from a reverse-phase (C-18) flash chromatographic column with 0.1% formic acid/MeCN (7:3).

**Scheme 2:**
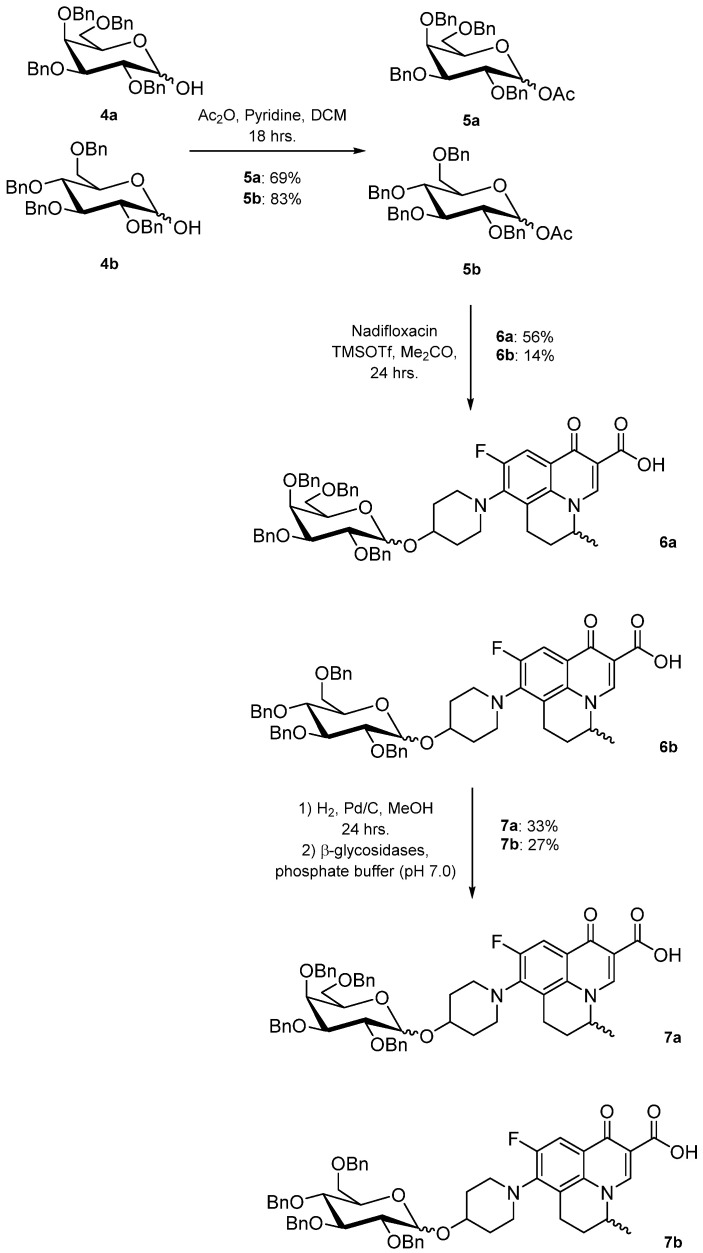
Synthesis and resolution of nadifloxacin-α-galactoside (**7a**) and α-glucoside (**7b**).

The *cis*-galactoside (**7a**) and *cis*-glucoside (**7b**) were obtained by changing the reaction solvent and glycoside-protecting groups to favour the formation of the thermodynamic product (Scheme 2). The tetra-*O*-benzyl glycosides (**4a**) and (**4b**) were acetylated with acetic anhydride, then coupled with nadifloxacin (**1**) using TMSOTf in anhydrous acetone. This afforded a mixture of glycoside anomers with α:β ratios of 1:1.59 for the galactoside (**6a**) and 1:1.23 for the glucoside (**6b**), as evidenced by ^1^H Nuclear Magnetic Resonance (NMR) spectroscopic analysis. The benzyl ethers were then removed by hydrogenolysis over palladium on charcoal to afford (**7a**) and (**7b**) as a mixture of anomers. These anomeric mixtures could not be separated by chromatography, and instead the β-glycoside was selectively hydrolysed using the corresponding β-glycosidase enzymes-β-galactosidase from *Escherichia coli* in phosphate-buffered saline pH 7.0 or β-glucosidase from almonds, in acetate buffer, pH 6.0. The mixtures were then purified using flash chromatography as described above. All intermediates (**2a-e**), (**5a-b**), (**6a-b**) and deprotected glycosides (**3a-e**) and (**7a,b**) were characterised by ^1^H and ^13^C NMR and IR, spectroscopic analysis and mass spectrometric analysis. Purity was determined by High Performance Liquid Chromatography (HPLC) to be > 97% prior to subsequent analysis of the inhibitory properties of the glycosides (**3a-e**) and (**7a,b**).

All of the synthesised nadifloxacin glycosides (**3a-e**) and (**7a,b**) were soluble in water at physiological pH as demonstrated in the final enzymatic resolution step in Scheme 2, wherein the product was dissolved in aqueous systems to allow for enzymatic hydrolysis. The octanol–water coefficients (Log P) were also calculated as 0.05 for the hexose glycosides and 0.51 for the pentoses, compared to 1.79 for underivatised nadifloxacin (**1**) (calculated using ChemDraw 12.0, Perkin-Elmer, Cambridge, UK).

It should be noted that racemic nadifloxacin was used in all glycoside syntheses, and the products (**3a-e**) and (**7a,b**) were isolated as mixtures of diastereomers. This afforded more complex NMR spectra than expected with some peaks unresolved and thus quoted as multiplets.

Since nadifloxacin (**1**) was developed as a potent antimicrobial for the treatment of MRSA, the antimicrobial activities of the synthesised nadifloxacin glycosides were determined for a range of *Staphylococcus aureas* strains. The lowest concentration at which bacterial growth could not be detected was recorded as the minimum inhibitory concentration (MIC). All of the *Staphylococcus aureas* strains were found to be sensitive to nadifloxacin, and all of the strains were found to be sensitive to, or intermediately sensitive to, the glycosides. However, all of the glycosides (**3a-e**) and (**7a,b**) had MICs that were higher than free nadifloxacin (**1**) (Table 1), presumably due to the requirement of hydrolysis to release the free nadifloxacin. This could indicate that the nadifloxacin glycosides do not induce the genes that produce the proteins required for transport and hydrolysis of the glycosides. There was variation in the MICs between glycosides and between strains for the same glycoside, probably due to differences in the rate of uptake of the glycoside by the bacteria and the amount of glycosidase that was expressed by the bacteria. Free nadifloxacin (**1**) was slightly more inhibitory for the MRSA strains than for non-MRSA organisms (0.125 μg/mL compared to 0.25–0.5 μg/mL), but there was no general pattern to the resistance of the glycosides. Of the glycosides, the α-L-arabinofuranoside (**3a**) was the most potent with MICs of 1–8 μg/mL for all of the strains that were examined.

## 3. Materials and Methods

NMR spectra were recorded on a Bruker DPX spectrometer (400 MHz), and chemical shifts are quoted in ppm relative to tetramethylsilane as internal standard using the following abbreviations: s, singlet, d, doublet, at, apparent triplet, as, apparent singlet and m, multiplet. Liquid Chromatography Mass Spectrometry (LCMS) was accomplished using a ThermoFisher Scientific Accela LC system coupled to a ThermoFisher Scientific LTQ Fleet Ion Trap Mass Spectrometer (ThermoFisher Scientific, Loughborough, UK). Melting points were recorded on a TA Instruments DSC Q2000 instrument heating at 10 °C/min (Hertfordshire, UK). FTIR spectra were recorded on a ThermoFisher Scientific Nicolet iS10 instrument. Thin-layer chromatography was performed using ALUGRAM SIL G precoated plates (Macherey-Nagel, Germany). The purity of deprotected samples was achieved using an Agilent 1100 series HPLC with a ThermoFisher Scientific Hypersil Gold Column (50 × 4.6 mm, 3 µm) eluting 0.1% (*v*/*v*) formic acid in water/MeCN (85:15), 2 mL/min, UV absorbance at 254 nm.

All culture media was “Oxoid” from ThermoFisher Scientific, Basingstoke, UK. Nadifloxacin was purchased from AOKChem (Shanghai, China) as a racemate and was used without further purification. Per-*O*-acetylated and 2,3,4,6-tetra-*O*-benzyl glucose and galactose were purchased from Carbosynth (Berkshire, UK). All other reagents were purchased from Sigma-Aldrich (Poole, Dorset, UK) or Fisher Scientific (Loughborough, UK).

**Synthesis of 1,2-*trans* glycosides promoted by 10 molar equivalents of TMSOTf–General method.**

Nadifloxacin (**1**) (5 g, 13.9 mmoles) was suspended in anhydrous acetonitrile (250 mL), and to this TMSOTf (2.51 mL, 13.9 mmoles) was added, and the solution was stirred. Following this were the additions of the per-*O*-acetylated glycoside (either 1,2,3,5-tri-*O*-acetyl-α-L-arabinofuranoside, 1,2,3,4,6-tetra-*O*-acetyl-β-D-galactopyranoside, 1,2,3,4,6-tetra-*O*-acetyl-β-D-glucopyranoside, 1,2,3,4,6-tetra-*O*-acetyl-α-D-mannopyranoside or 1,2,3,5-tri-*O*-acetyl-β-D-xylofuranoside, 13.9 mmoles) followed by TMSOTf (22.59 mL, 124.9 mmoles). Reactions were stirred at room temperature under an atmosphere of nitrogen for 24 h. The reaction mixtures were then diluted with anhydrous dichloromethane (250 mL), washed with sat. NaHCO_3_ (2 × 100 mL) and brine (2 × 100 mL), dried over MgSO_4_ and concentrated in vacuo. The products were purified on silica columns and eluted with toluene (A) and acetonitrile (B) at 14% B to 36% B over 5 column volumes and maintained at 36% for 10-column volumes.

**Nadifloxacin 2,3,5-tri-*O*-acetyl-α-** **L** **-arabinofuranoside (2a)**


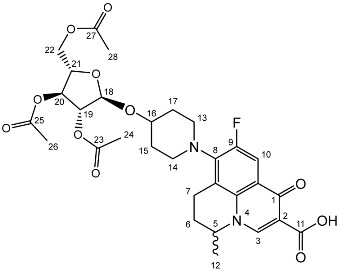


Nadifloxacin (**1**) (5 g, 13.9 mmoles) was added to anhydrous acetonitrile and reacted with 1 molar equivalent of TMSOTf, 1,2,3,5-tetra-*O*-acetyl-α-L-arabinofuranoside (4.53, 13.9 mmoles), followed by 9 molar equivalents of TMSOTf overnight to yield the product (**2a**) as a white powder.

Yield (4.81 g, 56%), m.p. 93–106 °C, ^1^H NMR (400 MHz, CDCl_3_) δ: 8.69 (1H, s, C3-H), 7.98 (1H, d, *J* = 12.5 Hz, C10-H), 5.25–5.21 (1H, m, C18-H), 5.13–5.08 (1H, m, C21-H), 5.02 (1H, d, *J* = 5.0 Hz, C19-H), 4.56 (1H, dd, *J* = 7.0, 3.5 Hz, C5-H), 4.44 (1H, dt, *J* = 11.5, 2.5 Hz, C22-Ha), 4.34–4.28 (1H, m, C20-H), 4.28–4.21 (1H, m, C22-Hb), 3.31 (1H, m, C7-Ha), 2.95–2.79 (1H, m, C7-Hb), 2.22–2.18 (2H, m, C6-H_2_), 2.16–2.09 (9H, m, acetyl CH_3_), 2.07–1.63 (4H, m, C15-H_2_ and C17-H_2_), 1.52 (3H, d, *J* = 6.5 Hz). ^13^C NMR (100 MHz, CDCl3) δ: 206.98 ketone (C=O), 170.62, 170.19, 169.84 (acetyl C=O), 167.26 (carbonyl C=O), 147.35 (C3), 137.83, 133.35 (aromatic C), 110.66 (C10), 107.63 (C18), 81.71 (C21), 80.32 (C20), 76.80 (C19), 63.29 (C22), 57.94 (C5), 32.71 (C15, C17), 30.91 (C16), 25.97 (C6), 21.44, 20.78, 20.75 (acetyl CH3), 20.20 (C5-CH3), 18.83 (C7). FT-IR ν cm^−1^: 2936.38 (w, carboxyl OH), 1738.96 (carboxylic or ester C=O), 1621.49 (aromatic C=C). MS +ESI found 619.2125 (MH^+^) C_30_H_36_FN_2_O_11_ required 619.2298.

**Nadifloxacin 2,3,4,6-tetra-*O*-acetyl-β-** **d** **-galactopyranoside (2b)**


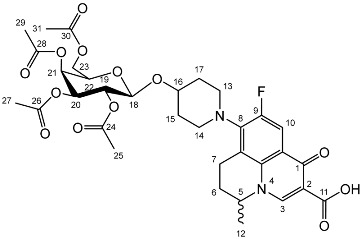


Nadifloxacin (**1**) (5 g, 13.9 mmoles) was added to anhydrous acetonitrile and reacted with 1 molar equivalent of TMSOTf, 1,2,3,4,6-penta-*O*-acetyl-β-D-galactopyranoside (5.6 g, 13.9 mmoles) followed by 9 molar equivalents of TMSOTf overnight to yield the product (**2b**) as a white powder.

Yield (4.98 g, 52%). m.p. 151–165 °C, ^1^H NMR (400 MHz, CDCl_3_) δ: 8.63 (1H, s, C3-H), 7.92 (1H, d, *J* = 12.0 Hz, C10-H), 5.39–5.31 (1H, m, C21-H), 5.19 (1H, at, *J* = 9.0 Hz, C19-H), 5.02–4.96 (1H, m, C20-H), 4.57 (1H, d, *J* = 8.0 Hz, C18-H), 4.53–4.45 (1H, m, C5-H), 4.15 (1H, dd, *J* = 11.5, 7.0 Hz, C23-Ha), 4.06 (1H, dd, *J* = 11.5, 7.0 Hz, C23-Hb), 3.87 (1H, at, *J* = 7.0 Hz, C22-H), 3.30–2.90 (5 H, m, C7-Ha, C13-H_2_, C14-H_2_), 2.79 (1 H, dt, *J* = 18.0, 8.5 Hz, C7-Hb), 2.14 (2H, m, C6-H_2_), 2.04 (2H, s, acetyl CH_3_), 2.01 (2H, s, acetyl CH_3_), 1.99 (3 H, s, acetyl CH_3_), 1.94 (3 H, s, acetyl CH_3_), 1.89–1.55 (4H, m, C15-H_2_, C17-H_2_), 1.46 (3H, d, *J* = 6.5 Hz, C12-H_3_). ^13^C NMR (100 MHz, CDCl_3_) δ: 170.41, 170.28, 170.19, 167.25 (carboxyl C=O), 146.35 (C3), 133.61 (aromatic C), 110.81, 110.59 (C10), 100.33 (C18), 70.86 (C20), 70.66 (C22), 68.98 (C19), 66.99 (C21), 61.23 (C23), 57.94 (C5), 25.97 (C6), 20.81, 20.71, 20.62 (Acetyl CH_3_), 20.23 (C12). FT-IR ν cm^−1^: 2976.02 (w, carboxyl OH), 1748.35 (s, ketone C=O), 1731.64 (carboxylic or ester C=O), 1624.15 (aromatic C=C). MS +ESI found 691.2141 (MH^+^) C_33_H_40_FN_2_O_13_ required 691.2509.

**Nadifloxacin 2,3,4,6-tetra-*O*-acetyl-β-** **d** **-glucopyranoside (2c)**


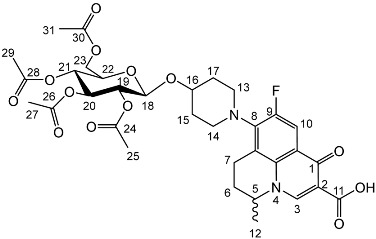


Nadifloxacin (**1**) (5 g, 13.9 mmoles) was added to anhydrous acetonitrile and reacted with 1 molar equivalent of TMSOTf, 1,2,3,4,6-penta-*O*-acetyl-β-D-glucopyranoside (5.6 g, 13.9 mmoles) followed by 9 molar equivalents of TMSOTf overnight to yield the product (**2c**) as a white powder.

Yield (2.73 g, 29%). m.p. 156–159 °C, ^1^H NMR (400 MHz, CDCl_3_) δ: 8.68 (1H, s, C3-H), 7.96 (1H, d, *J* = 12.0 Hz, C10-H), 5.22 (1H, dd, *J* = 9.5, 2.0 Hz, C20-H), 5.08 (1H, dd, *J* = 9.5, 2.0 Hz, C21-H), 5.04–4.96 (1H, m, C19-H), 4.67 (1H, d, *J* = 8.0 Hz, C18-H), 4.57 (1H, dt, *J* = 7.0, 3.5 Hz, C5-H), 4.25 (1H, dd, *J* = 12.5, 5.0 Hz, C23-Ha), 4.17–4.10 (1H, m, C23-Hb), 3.72 (1H, ddd, *J* = 10.0, 4.5, 2.5 Hz, C22-H), 3.33–2.94 (5 H, m, C7-Ha, C13-H_2_, C14-H_2_), 2.84 (1H, ddt, *J* = 17.0, 10.5, 5.5 Hz, C7-Hb), 2.15 (2H, m, C6-H_2_), 2.07 (3H, acetyl CH_3_), 2.04 (3H, acetyl CH_3_), 2.02 (3H, acetyl CH_3_), 2.00 (3H, acetyl CH_3_), 1.50 (3H, d, *J* = 6.5 Hz, C12-H_3_). ^13^C NMR (100 MHz, CDCl_3_) δ: 207.66 (C1), 170.78, 170.38, 169.51, 167.64 (carboxyl C=O), 146.45 (C3), 133.64 (aromatic C), 107.39 (C10), 99.17 (C18), 72.76 (C20), 71.71 (C22), 71.44 (C19), 68.43 (C21), 61.98 (C23), 57.99 (C5), 30.89 (C6), 20.1 (acetyl CH3), 20.14 (C12). FT-IR ν cm^−1^: 2973.23 (w, carboxyl OH), 1748.42 (s, ketone C=O), 1725.81 (carboxylic or ester C=O), 1672.21 (aromatic C=C). MS +ESI found 691.2930 (MH^+^) C_33_H_40_FN_2_O_13_ required 691.2509.

**Nadifloxacin 2,3,4,6-tetra-*O*-acetyl-α-** **d** **-mannopyranoside (2d)**


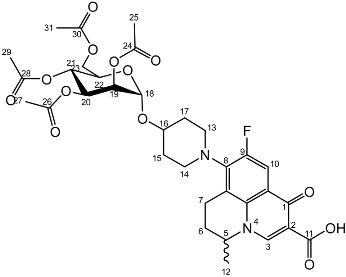


Nadifloxacin (**1**) (5 g, 13.9 mmoles) was added to anhydrous acetonitrile and reacted with 1 molar equivalent of TMSOTf, 1,2,3,4,6-penta-*O*-acetyl-D-mannopyranoside (5.6 g, 13.9 mmoles) followed by 9 molar equivalents of TMSOTf overnight to yield the product (**2d**) as a white powder.

Yield (2.20 g, 23%). m.p. 190–204 °C ^1^H NMR (400 MHz, CDCl3) δ: 8.63 (1H, s, C3-H), 7.92 (1H, d, *J* = 12.0 Hz, C10-H), 5.34 (1H, dd, *J* = 10.5, 3.0 Hz, C20-H), 5.24 (1H, dd, *J* = 10.0, 3.0 Hz, C21-H), 5.20–5.14 (1H, m, C19-H), 4.99–4.95 (1H, m, C18-H), 4.55–4.44 (1H, m, C5-H), 4.23 (1H, dd, *J* = 12.0, 4.5 Hz, C23-Ha), 4.12–4.01 (2H, m, C23-Hb, C22-H), 3.50–2.93 (3H, m, C7-Ha, C13-H_2_, C14-H_2_), 2.81 (1H, dt, *J* = 17.5, 9.5 Hz, C7-Hb), 2.11 (5H, m,C6-H2, acetyl CH_3_), 2.04 (acetyl CH_3_), 2.00 (acetyl CH_3_), 1.95 (acetyl CH_3_), 1.91–1.52 (4H, m, C15-H_2_, C17-H_2_) 1.47 (3H, d, *J* = 6.5 Hz, C12-H_3_). ^13^C NMR (100 MHz, CDCl_3_) δ: 177.36 (C11) 170.59, 170.22, 170.01, 169.72 (carboxyl C=O), 146.39 (C3), 133.65 (aromatic C), 110.79, 110.56 (C10), 107.66 (aromatic C) 95.97 (C18), 70.10 (C19), 69.01 (C20), 68.77 (C22), 66.31 (C21), 62.56 (C23), 57.96 (C5), 25.95 (C6), 20.95, 20.75, 20.73 (acetyl CH_3_), 20.24 (C12), 18.84 (C7). FT-IR ν cm^−1^: 2956.60 (w, carboxyl OH), 1737.23 (carboxylic or ester C=O), 1617.60 (aromatic C=C). MS +ESI found 691.2372 (MH^+^) C_33_H_40_FN_2_O_13_ required 691.2509.

**Nadifloxacin 2,3,5-tri-*O*-acetyl-β-** **d** **-xylofuranoside (2e)**


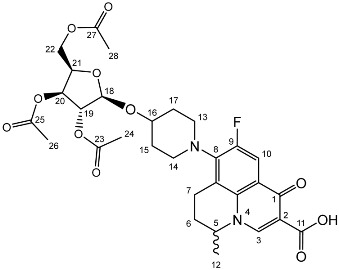


Nadifloxacin (**1**) (5 g, 13.9 mmoles) was added to anhydrous acetonitrile and reacted with 1 molar equivalent of TMSOTf, 1,2,3,5-tetra-*O*-acetyl-β-D-xylofuranoside (4.53, 13.9 mmoles) followed by 9 molar equivalents of TMSOTf overnight to yield the product (**2e**) as a white powder.

Yield (4.72 g, 55%). m.p. 84–95 °C, ^1^H NMR (400 MHz, CDCl3) δ: 8.70 (1H, s, C3-H), 7.98 (1H, d, *J* = 12.5 Hz, C10-H), 5.34 (1 H, dd, *J* = 6.0, 2.0 Hz, C20), 5.22–5.19 (1H, m, C18-H), 5.15–5.12 (1H, m, C19-H), 4.65–4.50 (2H, m, C5-H, C21-H), 4.37–4.11 (2H, m, C22-H_2_), 3.37–3.01 (5H, m, C7-Ha, C13-H2, C14-H_2_), 2.90 (1H, ddd, *J* = 18.0, 11.5, 7.0 Hz, C7-Hb), 2.23–1.69 (m, C6, acetyl CH_3_, C15-H_2_, C17-H_2_), 1.52 (3H, d, *J* = 7.0 Hz). ^13^C NMR (100 MHz, CDCl_3_) δ: 177.85 (C11), 170.78, 170.14, 169.94, 167.32 (acetyl C=O), 146.76 (C3), 138.33, 134.19 (aromatic C), 110.60, 110.36 (C10), 104.69 (C18), 81.38 (C19), 78.47 (C21), 75.27 (C20), 63.44 (C22), 58.49 (C5), 26.24 (C6), 21.00, 20.86, (acetyl C), 20.28 (C12), 19.25 (C13 or C14). FT-IR ν cm^−1^: 2936.38 (w, carboxyl OH), 1738.96 (carboxylic or ester C=O), 1621.49 (aromatic C=C). MS +ESI found 619.2062 (MH^+^) C_30_H_36_FN_2_O_11_ required 619.2298.

**Deprotection of per-*O*-acetylated nadifloxacin glycosides–General method**

The isolated glycosides (**2a-e**) were de-*O*-acetylated using a mixture of anhydrous dichloromethane and methanol (1:6) and potassium carbonate (50% *w*/*w*) over 30–60 min. The mixtures were adjusted to approximately pH 3 by the addition of formic acid and were purified on C18 columns and eluted with 0.1% (*v*/*v*) formic acid in water (A) and acetonitrile (B) isocratically at 7:3.

**Nadifloxacin α-** **l** **-arabinofuranoside (3a)**


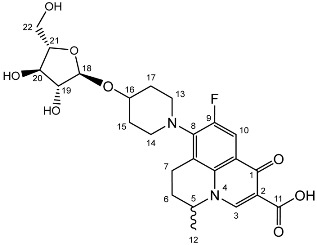


Nadifloxacin 2,3,5-tri-*O*-acetyl-α-L-arabinofuranoside (**2a**) (2.04 g, 3.31 mmoles) was dissolved in anhydrous dichloromethane and methanol, reacted with potassium carbonate (1.02 g) and purified to afford the product (**3a**) as an off-white powder.

Yield (1.12 g, 69%). mp: 167–178 °C, ^1^H NMR (400 MHz, DMSO-d6) δ: 8.94 (1H, s, C3-H), 7.81 (1H, d, *J* = 12.5 Hz, C10-H), 4.95–4.91 (1H, m, C18-H), 4.91–4.82 (1H, m, C5-H), 3.83–3.79 (1H, m, C19-H), 3.76 (1H, m, C21-H), 3.64 (1H, m, C20-H), 3.61–3.54 (1H, m, C22-Ha), 3.42 (1H, dd, *J* = 12.5, 6.0 Hz, C22-Hb), 3.37–3.07 (5H, m, C7-Ha, C13-H_2_ and C14-H_2_), 3.03–2.81 (1H, m, C7-Hb), 2.16–1.86 (3H, m, C6-H_2_, C15-H_2_ or C17-H_2_), 1.82–1.50 (2H, m, C15-H_2_ or C17-H_2_), 1.41 (3H, d, *J* = 6.5 Hz, C12). ^13^C NMR (100 MHz, DMSO-d6) δ: 206.32 (ketone C=O), 176.49, 166.35 (carboxyl C=O), 147.56 (C3), 133.81 (aromatic C), 109.06 (C10), 106.17 (C18), 83.54 (C21), 82.58 (C19), 77.09 (C20), 61.25 (C22), 57.10 (C5), 49.40 (C13 and C14), 33.70 (C15 or C17), 30.64 (C6), 19.62 (C12), 18.63 (C7). FT-IR ν cm^−1^: 3343.32 (m, alcohol OH), 2936.72 (w, carboxyl OH), 1716.43 (carboxylic or ester C=O), 1618.00 (aromatic C=C). MS +ESI found 493.1568 (MH^+^) C_24_H_30_FN_2_O_8_ required 493.1986. HPLC analysis: retention time: 5.912 min, 97.4%.

**Nadifloxacin β-** **d** **-galactopyranoside (3b)**


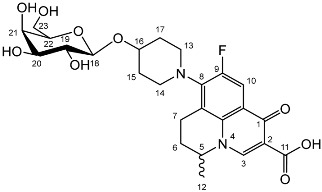


Nadifloxacin 2,3,4,6-tetra-*O*-acetyl-β-D-galactopyranoside (**2b**) (2 g, 2.90 mmoles) was dissolved in anhydrous dichloromethane and methanol, reacted with potassium carbonate (1 g) and purified to afford the product (**3b**) as an off-white powder.

Yield (1.12 g, 74%), m.p. 149–161 °C. ^1^H NMR (400 MHz, DMSO-d6) δ: 8.95 (1 H, d, *J* = 3.0 Hz, C3-H), 7.84 (1 H, dd, *J* = 12.5, 3.0 Hz, C10-H), 4.96–4.83 (1H, m, C5-H), 4.28–4.2 (1H, m, C18-H), 3.66–3.60 (1H, m, C20-H), 3.58–3.43 (2H, m, C23-H_2_), 3.44–3.20 (2H, m, C19-H, C21-H, C22-H), 3.11 (1H, d, *J* = 16.5 Hz, C7-Ha), 3.02–2.83 (1H, m, C7-Hb), 2.20–1.97 (2H, m, C6-H_2_), 2.02–1.53 (4H, m, C15-H_2_ and C17-H_2_), 1.41 (3H, d, *J* = 4.5 Hz, C12-H_3_). ^13^C NMR (100 MHz, DMSO-d6) δ: 166.08 (carboxyl C=O), 147.25 (C3), 133.46 (aromatic C), 108.99 (C10), 101.56 (C18), 75.09 (C22), 73.19, 70.39 (C19 and C21), 67.95 (C20), 60.45 (C23), 57.02 (C5), 33.66 (C15 and C17), 24.80 (C6), 19.55 (C12), 18.53 (C7). FT-IR ν cm^−1^: 3383 (w, alcohol C-OH), 2925 (w, carboxylic C-OH), 1698 (m, ketone C=O), 1628 (m, aromatic C=C), 1448 (m, alkyl C-H), 1021 (s, C-O). MS + ESI found 523.2897 (MH^+^) C_25_H_32_FN_2_O_9_ required 523.2092. HPLC analysis: retention time: 3.042 min, purity: 97.3%.

**Nadifloxacin β-** **d** **-glucopyranoside (3c)**


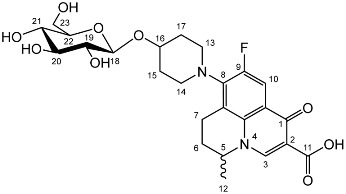


Nadifloxacin 2,3,4,6-tetra-*O*-acetyl-β-D-glucopyranoside (**2c**) (2.6 g, 3.77 mmoles) was dissolved in anhydrous dichloromethane and methanol, reacted with potassium carbonate (1.3 g) and purified to afford the product (**3c**) as an off-white powder.

Yield (1.26 g, 64%), m.p. 162–163 °C, ^1^H NMR (400 MHz, DMSO-d6) δ: 8.94 (1H, s, C3-H), 7.83 (1H, d, *J* = 12.0 Hz, C10-H), 4.93–4.80 (1H, m, C5-H), 4.30 (1H, d, *J* = 7.0 Hz, C18-H), 3.88 (2H, s, C13-H_2_ or C14-H_2_), 3.67 (1H, d, *J* = 11.5 Hz, C23-Ha), 3.43 (1H, d, *J* = 9.5 Hz, C23-Hb), 3.23 (2H, s, C13-H_2_ or C14-H_2_), 3.20–3.06 (4H, m, C20-H, C21-H, C22-H and C7-Ha), 2.96 (1H, at, *J* = 7.0 Hz, C19-H), 2.94–2.85 (1H, m, C7-Hb), 2.20–1.98 (2H, m, C6-H_2_), 1.85–1.48 (4H, m, C15-H_2_ and C17-H_2_), 1.41 (3H, d, *J* = 5.5Hz, C12-H_3_). ^13^C NMR (100 MHz, DMSO-d6) δ: 166.08 (carboxyl C=O), 147.10 (C3), 133.48 (aromatic C), 133.16 (aromatic C), 106.24 (C10), 100.41 (C18), 76.8 (C20, C22), 73.52 (C19), 70.45 (C21), 61.06 (C23), 57.04 (C5), 49 (C13 or C14) 33.60 (C15 and C16),19.56 (C12), 18.60 (C7). FT-IR ν cm^−1^: 3386 (w, b, alcohol C-OH), 2925 (w, carboxylic C-OH), 1703 (m, ketone C=O), 1628 (m, aromatic C=C), 1448 (m, alkyl C-H), 1021 (s, C-O). MS +ESI found 523.2073 (MH^+^) C_25_H_32_FN_2_O_9_ required 523.2092. HPLC analysis: retention time: 2.537 min, purity: 97.7%.

**Nadifloxacin α-** **d** **-mannopyranoside (3d)**


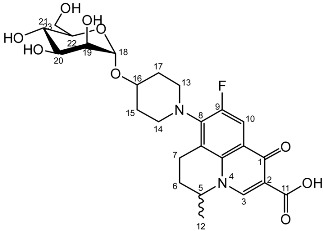


Nadifloxacin 2,3,4,6-tetra-*O*-acetyl-α-D-mannopyranoside (**2d**) (2.0 g, 2.90 mmoles) was dissolved in anhydrous dichloromethane and methanol, reacted with potassium carbonate (1.0 g) and purified to afford the product (**3d**) as an off-white powder.

Yield (1.10 g, 73%). m.p. 186 °C, ^1^H NMR (400 MHz, DMSO-d6) δ: 8.94 (1H, s, C3-H), 7.84 (1H, d, *J* = 12.5 Hz, C10-H), 4.92–4.81 (2H, m, C5-Hand C18-H), 3.83 (2H, as, C13-H_2_ or C14-H_2_), 3.70–3.62 (1H, m, C23-Ha), 3.62–3.58 (1H, m, C19-H), 3.55–3.30 (5H, m, C20-H, C21-H, C22-H, C23-Ha), 3.25–3.09 (1H, m, C7-Ha), 3.02–2.82 (1H, m, C7-Hb), 2.16–1.88 (4H, m, C6-H_2_ and C15-H_2_ or C17-H_2_), 1.80–1.52 (2H, m, C15-H_2_ or C17-H_2_), 1.46–1.35 (3H, m, C12-H_3_). ^13^C NMR (100 MHz, DMSO-d6) δ: 165.62 (aromatic C), 147.46 (C3), 133.75 (aromatic C), 109.03 (C10), 98.23 (C18), 74.27, 70.89, 70.77, 67.05 (C20, C21 and C22), 61.37 (C23), 56.95 (C5), 33.39 (C15 and C17), 19.58 (C12), 18.52 (C7). FT-IR ν cm^−1^: 3429.13, 3343.67 (m, alcohol OH), 2937.98 (w, carboxyl OH), 1722.74 (carboxylic or ester C=O), 1616.53 (aromatic C=C). MS +ESI found 523.2296 (MH^+^) C_25_H_32_FN_2_O_9_ required 523.2092. HPLC analysis: retention time: 6.923 min, purity: 98.1%.

**Nadifloxacin β-** **d** **-xylofuranoside (3e)**


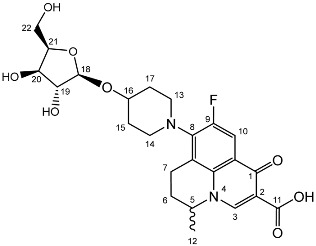


Nadifloxacin 2,3,5-tri-*O*-acetyl-β-D-xylofuranoside (**2e**) (2.31 g, 3.74 mmoles) was dissolved in anhydrous dichloromethane and methanol, reacted with potassium carbonate (1.16 g) and purified to afford the product (**3e**) as an off-white powder.

Yield (1.10 g, 66%). m.p.145 °C, ^1^H NMR (400 MHz, DMSO-d6) δ: 9.01 (1H, s, C3-H), 7.90 (1H, d, *J* = 12.5 Hz, C10-H), 5.00–4.91 (1H, m, C5-H), 4.34 (1H, d, *J* = 7.5 Hz, C18-H), 3.75 (1H, m, C22-Ha), 3.54–3.26 (1H, m, C21-H), 3.22–3.10 (3H, m, C20-H, C22-Hb, C7-Ha), 3.07–2.93 (2H, m, C19-H and C7-Hb), 2.26–1.92 (4H, m, C6-H_2_, C15-H_2_ or C17-H_2_), 1.87–1.61 (2 H, m, C15-H_2_ or C17-H_2_), 1.48 (3H, d, *J* = 4.0 Hz, C12-CH_3_). ^13^C NMR (100 MHz, DMSO-d6) δ: 147.79 (C3), 133.75 (aromatic C), 109.09 (C10), 102.3 (C18), 76.65 (C20), 73.28 (C19), 69.62 (C21), 65.65 (C22), 33.77 (C15 and C17), 24.91 (C6), 19.65 (C12), 18.85 (C7). FT-IR ν cm^−1^: 3335.48 (m, alcohol OH), 2933.13 (w, carboxyl OH), 1614.51 (aromatic C=C). MS + ESI found 493.1762 (MH^+^) C_24_H_30_FN_2_O_8_ required 493.1986. HPLC analysis: retention time: 8.713 min, purity: 98.7%.

**Synthesis of 1-*O*-acetyl-2,3,4,6-tetra-*O*-benzyl-** **d** **-glycosides (5a, 5b)–General method**

2,3,4,6-Tetra-*O*-benzyl-D-galactopyranose (**4a**) and 2,3,4,6-tetra-*O*-benzyl-D-glucopyranose (**4b**) (20 g, 37 mmoles) were each dissolved in anhydrous dichloromethane (100 mL). To these solutions pyridine (6.4 mL, 74.4 mmoles) and acetic anhydride (7.52 mL, 74.4 mmoles) were added. The mixtures were stirred under nitrogen, monitored by TLC (hexane–ether 1:1) and stirred at room temperature for approximately 18 h. The organic solutions were extracted with 1M HCl (3 × 100 mL), sat. NaHCO_3_ (3 × 100 mL) and brine (3 × 100 mL). The organic fractions were dried over MgSO_4_ and concentrated in vacuo to afford an oil. The galactoside (**5a**) was precipitated from methanol to afford a white powder. The glucoside (**5b**) was used as a crude oil.

**1-*O*-Acetyl-2,3,4,6-tetra-*O*-benzyl-** **d** **-galactopyranoside (5a)**


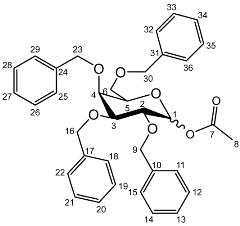
 Yield (14.8 g, 69%), m.p. 102–104 °C, isolated as a mixture of anomers, α:β 1:1.59, ^1^H NMR (400 MHz, CDCl_3_) δ: 7.38–7.22 (m, benzyl C-H), 6.38 (d, *J* = 3.5 Hz, α-C1-H), 5.57 (d, *J* = 8.0 Hz, β-C1-H), 4.95 (d, *J* = 11.5 Hz, α-benzyl CH_2_), 4.94 (d, *J* = 11.5 Hz, β-benzyl CH_2_), 4.84 (d, *J* = 11.5 Hz, β-benzyl CH_2_), 4.82 (d, *J* = 11.5 Hz, α-benzyl CH_2_), 4.74 (d, *J* = 11.5 Hz, α-benzyl CH_2_), 4.75–4.69 (m, benzyl CH_2_), 4.62 (d, *J* = 11.5 Hz, β-benzyl CH_2_), 4.57 (d, *J* = 11.5 Hz, α-benzyl CH_2_), 4.46 (d, *J* = 11.5 Hz, α-benzyl CH_2_), 4.43 (d, *J* = 11.5 Hz, β-benzyl CH_2_), 4.39 (d, *J* = 11.5 Hz, α-benzyl CH_2_), 4.38 (d, *J* = 11.5 Hz, β-benzyl CH_2_), 4.16 (dd, *J* = 10.0, 3.5 Hz, α-C2-H), 4.06–3.99 (m, C5-H), 3.94 (m, β-C2-H), 3.89 (dd, *J* = 10.0, 2.5 Hz, α-C3-H), 3.72–3.66 (m, β-C4-H), 2.11 (s, α-C8-H_3_), 2.03 (s, β-C8-H_3_). ^13^C NMR (100 MHz, DMSO-d6) δ: 169.63, 169.41 (carboxyl C=O), 138.64, 138.52, 138.42, 138.22, 138.06, 137.80, 137.77 (benzyl *ipso* C), 128.63–127.36 (benzyl CH), 94.30 (β-C1), 90.81 (α-C1), 82.42 (α-C5), 78.61 (α-C3), 78.19 (β-C2), 75.42 (α-C2), 75.35, 74.95, 74.72 (benzyl CH2), 74.08 (β-C4), 73.61, 73.54, 73.40, 73.40 (benzyl CH2), 73.04 (β-C5), 72.89 (benzyl CH2), 68.41 (α-C6), 67.94 (β-C6), 21.24 (α-C8), 21.06 (β-C8). FT-IR ν cm^−1^: 3059.70, 3026.72 (w, aromatic C-H), 1747.65 (m, ester C=O), 1081.32, 1045.51, 1024.29 (s, ether C-O). MS + ESI found 621.3333 (M + K^+^) C_36_H_38_KO_7_ required 621.2249.

**1-*O*-Acetyl-2,3,4,6-tetra-*O*-benzyl-** **d** **-glucopyranoside (5b)**


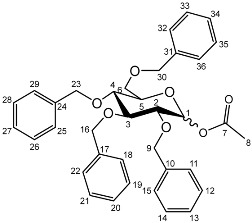
 Yield (18.0 g, 83%). Isolated as a mixture of anomers, α:β 1:1.23, ^1^H NMR (400 MHz, CDCl_3_) δ: 7.38–7.22 (m, benzyl C-H), 7.16–7.10 (m, benzyl C-H), 6.35 (d, *J* = 3.5 Hz, α-C1-H), 5.60 (d, *J* = 8.0 Hz, β-C1-H), 4.99–4.44 (m, benzyl CH2), 3.99–3.90 (m, glucose H), 3.89–3.83 (m, glucose H), 3.79–3.53 (m, α-, β-C2-H and α-, β-C6-H_2_), 2.13 (s, α-C8-H_3_), 2.05 (s, β-C8-H_3_). ^13^C NMR (100 MHz, DMSO-d6) δ: 169.47, 169.31 (C7), 138.64, 138.11, 138.02, 137.89, 137.82, 137.60 (aromatic C), 128.58–127.59 (aromatic C-H), 94.05 (α-C1), 84.82, 81.68, 81.07, 78.88, 77.22, 76.95 (glucose C), 75.72 (benzyl CH_2_), 75.40 (glucose C), 75.30, 75.04, 73.55, 73.22 (benzyl CH_2_), 72.83 (glucose C), 68.09 (C6), 21.10 (C8). FT-IR ν cm^−1^: 3088.08, 3062.95, 3030.18 (w, aromatic C-H), 1755.44 (m, ester C=O), 1072.79, 1027.24 (s, ether C-O). MS-ESI found 621.5834 (M + K^+^) KC36H38O7 required 621.2249.

**Synthesis of benzyl protected nadifloxacin α-galactoside (6a) and α-glucoside (6b)–General method**

Nadifloxacin (**1**) (5 g, 13.9 mmoles) was suspended in anhydrous acetone (250 mL), and to this TMSOTf was added (2.51 mL, 13.9 mmoles), and the solution was stirred. To this the 1-*O*-acetyl-2,3,4,6-tetra-*O*-benzyl-glycoside (**5a** or **5b**) (16.1 g, 13.9 mmoles) was added followed by TMSOTf (22.59 mL, 124.9 mmoles). Reactions were stirred at room temperature under an atmosphere of nitrogen for 24 h. The reaction mixtures were then diluted with anhydrous dichloromethane (250 mL), washed with sat. NaHCO_3_ (2 × 100 mL) and brine (2 × 100 mL), dried over MgSO_4_ and concentrated in vacuo. The products (**6a**) or (**6b**) were purified on silica columns and eluted with toluene (A) and acetonitrile (B) at 14% B to 36% B over 5 column volumes and maintained at 36% for 10-column volumes.

**Nadifloxacin 2,3,4,6-tetra-*O*-benzyl-α,β-** **d** **-galactopyranoside (6a)**


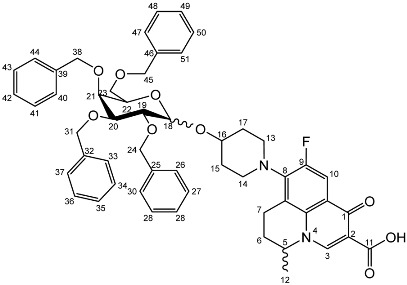
 Yield (6.83 g, 56%). m.p. 61–65 °C, isolated as a mixture of anomers, α:β 3:5, ^1^H NMR (400 MHz, CDCl_3_) δ: 8.63 (1H, s, C3-H), 7.93 (1H, d, *J* = 12.0 Hz, C10-H), 7.36–7.15 (m, benzyl C-H), 4.98–4.94 (m, α-C18-H), 4.94–4.84 (m, benzyl CH_2_), 4.83–4.46 (m, benzyl CH_2_, C5-H), 4.44 (d, *J* = 8.5 Hz, β-C18-H), 4.41–4.30 (m, benzyl CH_2_), 4.03–3.95 (m, α-C19-H and C22-H), 3.95–3.88 (C21-H), 3.82 (d, *J* = 9.5 Hz, C22-H), 3.77 (d, *J* = 9.0 Hz, β-C19-H), 3.52–3.44 (m, C20-H and C23-H_2_), 3.21 (d, *J* = 17.0 Hz, C7-Ha), 3.03–2.90 (m, C16-H), 2.85–2.67 (m, C7-Hb), 2.15–2.03 (m, C6-H_2_), 2.03–1.59 (m, C15-H_2_ and C17-H_2_), 1.48–1.39 (m, C12-H_3_). ^13^C NMR (100 MHz, CDCl_3_) δ: 167.16 (carboxyl C=O), 146.47 (C3), 128.62–127.27 (aromatic C), 125.29 (aromatic C), 110.72 (C10), 101.92 (β-C18), 5.83 (α-C18), 82.12 (C20), 79.77 (β-C19), 79.27 (C21), 76.06 (α-C19), 75.32 (benzyl CH2), 75.03 (C21), 74.77 (benzyl CH_2_), 74.53 (benzyl CH_2_), 73.94 (C20), 73.65 (C22) 73.13 (benzyl CH_2_), 69.78 (C22), 69.31, 69.01 (C23), 32.64 (C15, C17), 26.04 (C6), 20.25 (C12), 18.93 (C7). FT-IR ν cm^−1^: 3061.62, 3029.55 (w, aromatic C-H), 2920.54, 2861.87 (w, carboxylic C-OH), 1723.88 (m, carbonyl C=O), 1619 (m, C=C), 1490 (s, arene C-C), MS +ESI found 883.3593 (MH^+^) C_53_H_56_FN_2_O_9_ expected 883.3970.

**Nadifloxacin 2,3,4,6-tetra-*O*-benzyl-α,β-** **d** **-glucopyranoside (6b)**


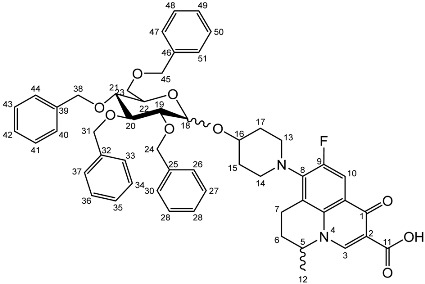
 Yield (1.75 g, 14%), ^1^H NMR (400 MHz, CDCl3) δ: 8.69 (s, C3-H), 8.00 (d, *J* = 12.0 Hz, C10-H), 7.41–7.21 (m, benzyl H), 5.06–4.96 (m, α-C18-H, benzyl CH_2_), 4.95–4.72 (m, benzyl CH_2_), 4.71–4.44 (benzyl CH_2_, β-C18-H, C5-H), 4.03 (at, *J* = 9.5 Hz, α-C20-H), 3.94–3.87 (m, glucose H), 3.78–3.71 (m, C23-Ha), 3.71–3.44 (m, C23-Hb, α-C19-H, β-C19-H, glucose H), 3.38–3.16 (m, C7-Ha), 3.15–3.00 (m, C13-H_2_ or C14-H_2_), 2.93–2.75 (m, C7-Hb), 2.11–1.64 (m, C17-H_2_, C15-H_2_), 1.53 (d, *J* = 6.5 Hz, C12-H_3_), 1.52 (d, *J* = 6.5 Hz, C12-H_3_).^13^C NMR (100 MHz, CDCl_3_) δ: 177.14, 167.35 (carbonyl C=O), 110.77, 110.49 (C10), 101.88 (β-C18), 95.13 (α-C18), 84.52, (glucose C), 82.29 (β-C19), 82.04 (α-C20), 79.98, 77.88 (glucose C), 75.69, 75.25, 75.04, 74.95 (benzyl CH_2_), 74.82 (β-glucose C), 73.50, 73.42, 73.33, 73.25 (benzyl CH2), 70.51 (glucose C), 68.62 (C23), 57.95 (C5), 33.12 (C17, C15), 30.97 (C6), 20.23 (C12), 18.83 (C7). FT-IR ν cm^−1^: 3062.43, 3028.98 (w, aromatic C-H), 2900.68, 2865.08 (w, carboxylic C-OH), 1618.42 (m, C=C), 1452 (s, arene C-C), 1067.21, 1027.67 (s, C-O). MS + ESI found 883.3850 (MH^+^) C_53_H_56_FN_2_O_9_ expected 883.3970.

**Deprotection of per-*O*-benzylated nadifloxacin glycosides and resolution of anomers by enzyme hydrolysis (7a, 7b)–General method**

De-*O*-benzylation of glycosides (**6a**) and (**6b**) was achieved using Pd/C (10 wt.%) in methanol under an atmosphere of hydrogen. After 24 h, the mixtures were filtered over Celite and concentrated in vacuo to afford anomeric mixture of nadifloxacin glycosides (**7a** α,β) and (**7b** α,β). These were then stirred in a suitable buffer, β-glycosidase (100 U) was added for **7a** α,β: β-galactosidase from *E. coli*, for **7b** α,β, β-glucosidase from almonds, and the suspensions were incubated at 37 °C and monitored by HPLC analysis for the decrease in the β-glycoside. Once the β-anomers had all been hydrolysed, the reaction mixtures were filtered, adjusted to pH 3.0 using formic acid and purified on C18 columns and eluted with 0.1% (*v*/*v*) formic acid in water (A) and acetonitrile (B) (70:30).

**Nadifloxacin α-** **d** **-galactopyranoside (7a)**


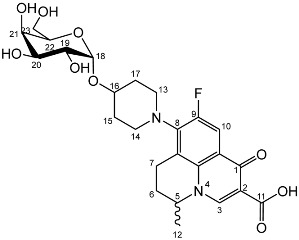
 Yield (1.25 g, 33%), m.p. 143–150 °C, ^1^H NMR (400 MHz, DMSO-d6) δ: 8.94 (1H, s, C3-H), 7.83 (1H, d, *J* = 12.0 Hz, C10-H), 4.99–4.76 (2H, m, C18-H and C5-H), 3.76–3.67 (3H, galactose C-H), 3.63–3.55 (2H, m, C19-H and galactose C-H), 3.54–3.40 (2H, m, C23-H_2_), 3.47–3.17 (4H, m, C13-H_2_ and C14-H_2_), 3.03–2.83 (2H, m, C7-H_2_), 2.21–196 (2H, m, C6-H_2_), 2.00–1.54 (4H, m, C15-H_2_ and C17-H_2_), 1.41 (3H, d, *J*= 5.0 Hz, C12-H_3_). ^13^C NMR (100 MHz, DMSO-d6) δ: 166.08 (Carboxyl C=O), 147.25 (C3), 133.50 (Aromatic C), 108.87 (C10), 106.28 (Aromatic C), 97.64 (C18), 71.45, 69.55, 68.87, 68.24 (galactose C), 60.57 (C23), 57.06 (C5), 33.08 (C15 and C17), 24.76 (C7), 19.57 (C12), 18.51 (C6). FT-IR ν cm^−1^: 3373.83 (m, alcohol OH), 2934.33 (w, carboxyl OH), 1708.29 (carboxylic or ester C=O), 1620.84 (aromatic C=C). MS + ESI found 523.2216 (MH^+^) C_25_H_32_FN_2_O_9_ required 523.2092. HPLC analysis: retention time: 4.127 min, purity: 98.2%.

**Nadifloxacin α-** **d** **-glucopyranoside (7b)**


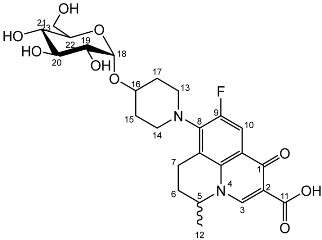
 Yield (0.20 g, 27%), m.p. 162 °C, ^1^H NMR (400 MHz, DMSO-d6) δ: 9.01 (1H, s, C3-H), 7.90 (1H, d, *J* = 12.0 Hz, C10-H), 5.03–4.83 (2H, m, C5-H and C18-H), 3.69 (1H, d, *J* = 11.0 Hz, C23-Ha), 3.61–3.46 (3H, m, C20-H, C22-H, C23-Hb), 3.30–3.20 (1H, m, C19-H), 3.25–3.12 (1H, m, C6-Ha), 3.20–3.08 (1H, m, C21-H), 3.07–2.88 (1H, m, C6-Hb), 2.25–1.95 (2H, m, C7-H2), 2.07–1.60 (4H, m, C15-H_2_ and C17-H_2_), 1.48 (3H, d, *J*= 7.0 Hz). ^13^C NMR (100 MHz, DMSO-d6) δ: 176.47 (carboxyl C=O), 166.08 (aromatic C), 147.40 (C3), 133.61 (aromatic C), 108.99 (C10), 106.44 (aromatic C), 97.28 (C18), 73.16 (C20), 73.05 (C22), 71.81 (C19), 70.37 (C21), 61.03 (C23), 57.07 (C5), 24.84 (C7), 19.57 (C12), 18.53 (C15 and C17). FT-IR ν cm^−1^: 3359.86 (m, alcohol OH), 2928.52 (w, carboxyl OH), 1709.04 (carboxylic or ester C=O), 1621.75 (aromatic C=C). MS + ESI found 523.1920 (MH^+^) C_25_H_32_FN_2_O_9_ required 523.2092. HPLC analysis: retention time: 5.028 min, purity 98.4%.

**Determination of minimum inhibitory concentration**

The glycosides (**3a-e** and **7a,b**) and underivatised nadifloxacin (**1**) were individually dissolved in dimethyl sulfoxide, and amounts of the solutions were added to Tryptone Soya Agar (TSA) that had been autoclaved and then cooled to 50 °C to produce doubling concentrations of nadifloxacin (**1**) from 0.125–8 μg/mL and 1–128 μg/mL of the glycosides (**3a-f** and **7a,b**). The agar was swirled to mix, and then plates were poured and dried. Specifically, a stock of nadifloxacin (**1**) was prepared in DMSO (1.6 mg/mL). Concentrations of 800, 400, 200, 100, 50 and 25 μg/mL were prepared by serial dilutions starting with addition of 0.5 mL of the 1.6 mg/mL solution into 0.5 mL of DMSO and so forth. An aliquot (100 μL) from each concentration was added individually to molten agar (20 mL) to give final agar concentrations of 8, 4, 2, 1, 0.5, 0.25 and 0.125 μg/mL. A stock of each nadifloxacin glycoside was prepared in DMSO (25.6 mg/mL). Concentrations of 12.8, 6.4, 3.2, 1.6, 0.8, 0.4 and 0.2 mg/mL were prepared by serial dilutions starting with addition of 0.5 mL of the 25.6 mg/mL solution into 0.5 mL of DMSO. A nadifloxacin glycoside concentration of 20 μg/mL was prepared by diluting 100 μL of concentration 0.2 mg/mL into 900 μL of DMSO. An aliquot (100 μL) from each concentration was added individually to molten agar (20 mL) to give final agar concentrations of 128, 64, 32, 16, 8, 4, 2, 1 and 0.1 μg/mL. A plate containing no nadifloxacin glycoside was used as control. Plates were cooled and dried before use. A plate containing no nadifloxacin was used as control. Plates were cooled and dried before use.

Cultures of bacteria were grown overnight in Nutrient Broth 2 (NB2) at 37 °C from beads stored at −80 °C. Cultures were then diluted decimally in Maximum Recovery Diluent (MRD) to approximately 10^6^ CFU/mL, and 300 µL of each organism added to the wells of a multipoint inoculator (“Oxoid Cathra Replicator”, Thermofisher Scientific). Pins of the inoculator then dispensed amounts of bacterial suspension onto the surface of the agar plates. Plates were incubated at 37 °C and inspected after 18 h for growth to obtain cultures in the log phase, wherein cells were actively dividing. The lowest amount of antibacterial agent that totally inhibited growth was recorded as the MIC for that organism.

## 4. Conclusions

The poor aqueous solubility of nadifloxacin (**1**) has limited its clinical use to topical applications, and this is unfortunate since it exhibits broad-spectrum antibacterial activity at very low concentrations. The glycosides prepared in this study were water-soluble, meaning that aqueous-based preparations are now feasible. Importantly, all of the *Staphylococcus aureus* strains, including four MRSA strains, were found to be sensitive to, or intermediately sensitive to, the glycosides. Hence, the glycosides could be suitable as lead compounds for the development of orally administered antibacterial agents for the treatment of MRSA infections. The stability of the glycosidic bond should also allow them to arrive at the site of infection intact. The most potent glycoside, nadifloxacin-α-L-arabinofuranoside (**3a**), had MICs of 1–8 μg/mL for the eight *S. aureus* strains, making it a prime candidate for further studies. Since the glycosides exhibited different MICs for different organisms, they could also be developed as tools for the selective isolation of target organisms in the presence of background flora.

## Figures and Tables

**Table 1:** MICs (μg/mL) for nadifloxacin (**1**) and synthesised glycosides (**3a-e**) and (**7a**,**b**) against drug-susceptible and -resistant *S. aureus* in TSA.

Species	Strain	1	3a	3b	3c	3d	3e	7a	7b
*S. aureus*	ATCC 6538	0.25	2	8	64	16	2	8	16
	ATCC 6538p	0.25	2	8	128	16	1	64	16
	ATCC 9144	0.5	1	8	32	8	1	4	16
	ATCC 25923	0.25	2	8	64	16	2	8	32
*S. aureus* (MRSA)	ATCC 33591	0.125	1	32	64	128	1	2	4
	ATCC 43300	0.125	2	2	4	8	32	64	128
	NCTC 10442	0.125	8	2	2	4	32	2	128
	NCTC 12493	0.125	2	32	8	64	32	32	32

**1:** nadifloxacin, **3a**: α-arabinoside, **3b**: β-galactoside, **3c**: β-glucoside, **3d**: α-mannoside, **3e**: β-xyloside, **7a**: α-galactoside, **7b**: α-glucoside.

## Data Availability

Not applicable.

## Sample Availability

Not available.

## References

[1] Coates R., Moran J., Horsburgh M.J. (2014). Staphylococci: Colonizers and pathogens of human skin. Future Microbiol..

[2] Otto M. (2010). Staphylococcus colonization of the skin and antimicrobial peptides. Expert Rev. Dermatol..

[3] Bergin S.P., Holland T.L., Fowler V.G., Tong S.Y.C. (2017). Bacteremia, Sepsis, and Infective Endocarditis Associated with *Staphylococcus aureus*. Curr. Top Microbiol. Immunol..

[4] Lowy F.D. (1998). Staphylococcus aureus infections. N. Engl. J. Med..

[5] Benner E.J., Kayser F.H. (1968). Growing clinical significance of methicillin-resistant Staphylococcus aureus. Lancet.

[6] Stewart G.T., Holt R.J. (1963). Evolution of natural resistance to the newer penicillins. Br. Med. J..

[7] Ploy M.C., Grelaud C., Martin C., de Lumley L., Denis F. (1998). First clinical isolate of vancomycin-intermediate Staphylococ-cus aureus in a French hospital. Lancet.

[8] Alba U.E., Angeles Dominguez M., Nagy E., Nord C.E., Palacín C., Vila J. (2009). In vitro activity of nadifloxacin against several Gram-positive bacteria and analysis of the possible evolution of resistance after 2 years of use in Germany. Int. J. Antimicrob. Agents..

[9] Nenoff P., Haustein U.-F., Hittel N. (2004). Activity of nadifloxacin (OPC-7251) and seven other antimicrobial agents against aerobic and anaerobic Gram-positive bacteria isolated from bacterial skin infections. Chemotherapy.

[10] Narayanan V., Motlekar S., Kadhe G., Bhagat S. (2014). Efficacy and safety of nadifloxacin for bacterial skin infections: Results from clinical and post-marketing studies. Derm. Ther..

[11] Vogt K., Hermann J., Blume U., Gollnick H., Hahn H., Haustein U.F., Orfanos C.E. (1992). Comparative activity of the topical quinolone OPC-7251 against bacteria associated with acne vulgaris. Eur. J. Clin. Microbiol. Infect. Dis..

[12] Cheng Y., Qu H., Ma M., Xu Z., Xu P., Fang Y., Xu T. (2007). Polyamidoamine (PAMAM) dendrimers as biocompatible carriers of quinolone antimicrobials: An in vitro study. Eur. J. Med. Chem..

[13] Shinde U., Pokharkar S., Modani S. (2012). Design and evaluation of microemulsion gel system of nadifloxacin. Indian J. Pharm. Sci..

[14] de Souza N.J., Shrikant V., Gupte S.V., Deshpande P.K., Desai I.N., Bhawsar S.B., Yeole R.D., Shukla M.C., Strahilevitz J., Hooper D.C. (2005). A chiral benzoquinolizine-2-carboxylic acid arginine salt. J. Med. Chem..

[15] Patel M.V., Agarwal S., Kandepu S., Shetty N., Upadhyay D.J., Chaturvedi N.C., Thomas A., Souza N.J.D., Khorakiwala H.F. (2004). Antibacterial Optically Pure Benzoquinolizine Carboxylic Acids, Processes, Compositions and Methods of Treatment.

[16] Kytidou K., Artola M., Overkleeft H.S., Aerts J.M. (2020). Plant glycosides and glycosidases: A treasure-trove for therapeutics. Front. Plant Sci..

[17] Křen V., Řezanka T. (2008). Sweet antibiotics–The role of glycosidic residues in antibiotic and antitumor activity and their randomization. FEMS Microbiol. Rev..

[18] Howse G.L., Bovill R.A., Stephens P.J., Osborn H.M.I. (2019). Synthesis and antibacterial profiles of targeted triclosan derivatives. Eur. J. Med. Chem..

[19] Bovill R.A., Evans P.G., Howse G.L., Osborn H.M.I. (2016). Synthesis and biological analysis of novel glycoside derivatives of L-AEP, as targeted antibacterial agents. Bioorg. Med. Chem. Lett..

[20] Cellier M., Fazackerley E., James A.L., Orenga S., Perry J.D., Turnbull G., Stanforth S.P. (2014). Synthesis of 2-arylbenzothiazole derivatives and their application in bacterial detection. Bioorg. Med. Chem..

[21] Sanderson T.J., Black C.M., Southwell J.W., Wilde E.J., Pandey A., Herman R., Thomas G.H., Boros E., Duhme-Klair A.-K., Routledge A. (2020). A Salmochelin S4-Inspired Ciprofloxacin Trojan Horse Conjugate. ACS Infect. Dis..

